# Relationships between altitude and depressive symptoms among middle-aged and older adults in China: a longitudinal study from the China health and retirement longitudinal study

**DOI:** 10.3389/fpsyt.2024.1436541

**Published:** 2024-10-15

**Authors:** Qing Ma, Wei Jiang, Qiuyan Zhao, Xin Xia, Ronghua Fang

**Affiliations:** ^1^ General Practice Ward, International Medical Center Ward, General Practice Medical Center, West China Hospital/West China School of Nursing, Sichuan University, Chengdu, China; ^2^ The Center of Gerontology and Geriatrics and National Clinical Research Center for Geriatrics, West China Hospital, Sichuan University, Chengdu, China

**Keywords:** altitude, depressive symptoms, middle-aged and older adults, Chinese, longitudinal study 1

## Abstract

**Background:**

Previous studies have consistently documented positive associations between depressive symptoms and altitude; however, a longitudinal study of these relationships among middle-aged and older adult community populations in China has not been previously reported.

**Methods:**

We screened 17,705 subjects who met the inclusion criteria from the China Health and Retirement Longitudinal Study database 2011. The altitude is the altitude at which the participants lived in our study area. We estimated the prospective associations between altitude and depressive symptoms among middle-aged and older adults. The 10-item Center for Epidemiological Studies Depression Scale short form was used to measure depressive symptoms. A total of 6,594 participants without depressive symptoms were recruited from the same cohort in 2011 and were followed up in 2018. Multivariate logistic regression was used to assess the associations between altitude and depressive symptoms among middle-aged and older adults.

**Results:**

The prevalence of depressive symptoms was 27.3% in our study. The prevalence of depressive symptoms increased with increasing altitude, and the prevalence of depressive symptoms in women was greater than that in men. Multivariate logistic regression adjusted for all other relevant variables showed that sex, altitude, education level, professional status and marital status were associated with depressive symptoms.

**Conclusions:**

This finding provides evidence of the relationship between altitude and depressive symptoms among middle-aged and older adult community populations in China and shows that depressive symptoms are significantly positively correlated with altitude and other factors, including sex, education level, professional status, and marital status.

## 1 Background

China is the most populous country in the world. Owing to the one-child policy and unprecedented social changes over the past few decades, China’s aging problem has become more serious than that of any other country in the world in recent years (1, 2). Depressive symptoms are the most common mental illness among middle-aged and older adults and are characterized by a high incidence, high suicide rate, low identification rate, low diagnosis rate and low treatment rate (3). Depressive symptoms not only deteriorate the health of middle-aged and older adults but also lead to cognitive dysfunction, worsening of chronic diseases, disability, and even suicide (4–7). However, the resources of mental health services in China are still insufficient to meet the growing needs of the general population (8). The prevalence of depressive symptoms in middle-aged and senior people in the United States, Europe, and Korea is 9.2%, 20.3% and 19.2%, respectively (9–11). The prevalence of depressive symptoms in middle-aged and elderly people varies widely in China, ranging from 8.02%-24.1% (3, 12). Therefore, depressive symptoms have become an urgent health problem in China.

Psychosocial, biological, hereditary, and environmental factors influence depressive symptoms (13, 14). Previous studies have shown that environmental factors (such as altitude) can negatively affect mood (15–17). In high-altitude areas, people undergo a series of biological changes, such as hypobaric hypoxia, which can reduce serotonin utilization, change dopamine levels, and increase inflammatory responses (18). These changes generate alterations in the brain structure (cerebellum, brain stem and olfactory cortex) (19), which results in depressive symptoms. Conversely, high altitudes, cold climates, strong winds, high levels of precipitation and altered circadian cycles may lead to altered hippocampal neurogenesis and decreased tryptophan hydroxylase 2 activity, which affects serotonin metabolism and reduces the synthesis of 5-hydroxytryptophan (5-HTP), thus decreasing serotonin levels within the central nervous system (20). The level of serotonin in the nervous system is an important neurotransmitter associated with mood, and low serotonin levels can lead to depressive symptoms (20), thereby increasing suicidal behavior (21).

A previous study reported that the prevalence of depressive symptoms among elderly people living in high-altitude (average altitude of 2300 meters above sea level, masl) nursing homes was almost five times greater (59.4% *vs* 11.1%) than that among those living in low-altitude areas (average altitude of 10 masl) in China (22). Another study revealed that 28.6% of middle-aged and older adults with depressive symptoms (CESD-10 scores ≥ 14) reside on the Qinghai-Tibet Plateau (average altitude: > 3,000 masl) (16), which is greater than that reported in a Chinese study and Western study (13.2% and 17.6%, respectively) (23, 24). Notably, because high-altitude nursing homes have poor social welfare, low pensions after retirement and a high-altitude environment limit the popularity of psychological counseling, which leads to a high prevalence of depressive symptoms. However, a study conducted at altitudes ranging from 3,600-4,800 masl reported a lower prevalence of depressive symptoms (1.8-2.9%) among residents of Tibet and the Andes, suggesting that there may be other influential sociocultural factors, such as religion and social family ties, may even inhibit the development of depressive symptoms in populations living at high altitudes (25). Overall, the differences in the prevalence of depressive symptoms among middle-aged and older adults at high altitudes may be related to demographic and clinical characteristics, small sample sizes, the lack of a random sample, the lack of a control group, the use of different measurement tools, and differences in altitude and depressive symptom cutoff points. Therefore, comparisons of the results of these studies should be carried out with caution.

Studies have also reported that high-altitude exposure can also cause breathing difficulties, headaches, sleep disturbances, chronic respiratory diseases, and health problems that can worsen depressive symptoms in middle-aged and older adults (26–28). A study from Saudi Arabia revealed that the prevalence of suicidal ideation in depressed patients at 2,400 masl was significantly greater than that at lower altitudes (sea level) (11.6% *vs* 2.1%) (26). The research results of Korean scholars are consistent with those of Saudi Arabia, suggesting that the suicide rate increased by 1.5% for every meter increase in average altitude (29). However, the factors influencing depressive symptoms among middle-aged and elderly individuals in high-altitude areas are complex. Therefore, comparisons of the results of these studies should be carried out with caution. Research on depressive symptoms in middle-aged and elderly people at high altitudes is limited, and most of these studies were cross-sectional and could not explain causality.

A longitudinal study was used to explore the relationships between exposure factors and disease outcomes in this study. To our knowledge, there is no longitudinal study on the relationship between depressive symptoms and altitude among middle-aged and elderly community residents in China. Therefore, we used data from the China Health and Retirement Longitudinal Study (CHARLS) to explore the correlation between altitude and depressive symptoms among middle-aged and elderly community residents in China, with the aim of increasing people’s attention to depressive symptom management and improving the health status of middle-aged and elderly community residents in high-altitude areas.

## 2 Methods

### 2.1 Study population and design

The CHARLS is a nationally representative longitudinal survey of Chinese people aged 45 years or older in mainland China (including 28 provinces, municipal cities, and autonomous regions) and their spouses, including assessments of the social, economic, and health status of community residents. A multistage stratified sampling design was used to ensure the representativeness of the sample. In the CHARLS 2011, a total of 17,705 participants were recruited. All of the participants were followed up every 2 years after the baseline survey. The CHARLS data can be accessed through its official website (charls.ccer.edu.cn/en).

In our study, we used data from the CHARLS 2011 and 2018 and analyzed altitude and depressive symptom outcomes in 2018. According to the purpose of this research, we formulated the following inclusion criteria for the study subjects: (1) ≥ 45 years of age or older, as well as demographic data such as sex, education level, marital status, residence, smoking status, drinking status, height and weight; and (2) data regarding the 10-item Center for Epidemiological Studies depressive symptoms scale (CESD-10) score. The exclusion criteria were (1) no information about age; (2) lacking values for the main variables; and (3) having a baseline CESD-10 score > 10.

**Figure 1:**
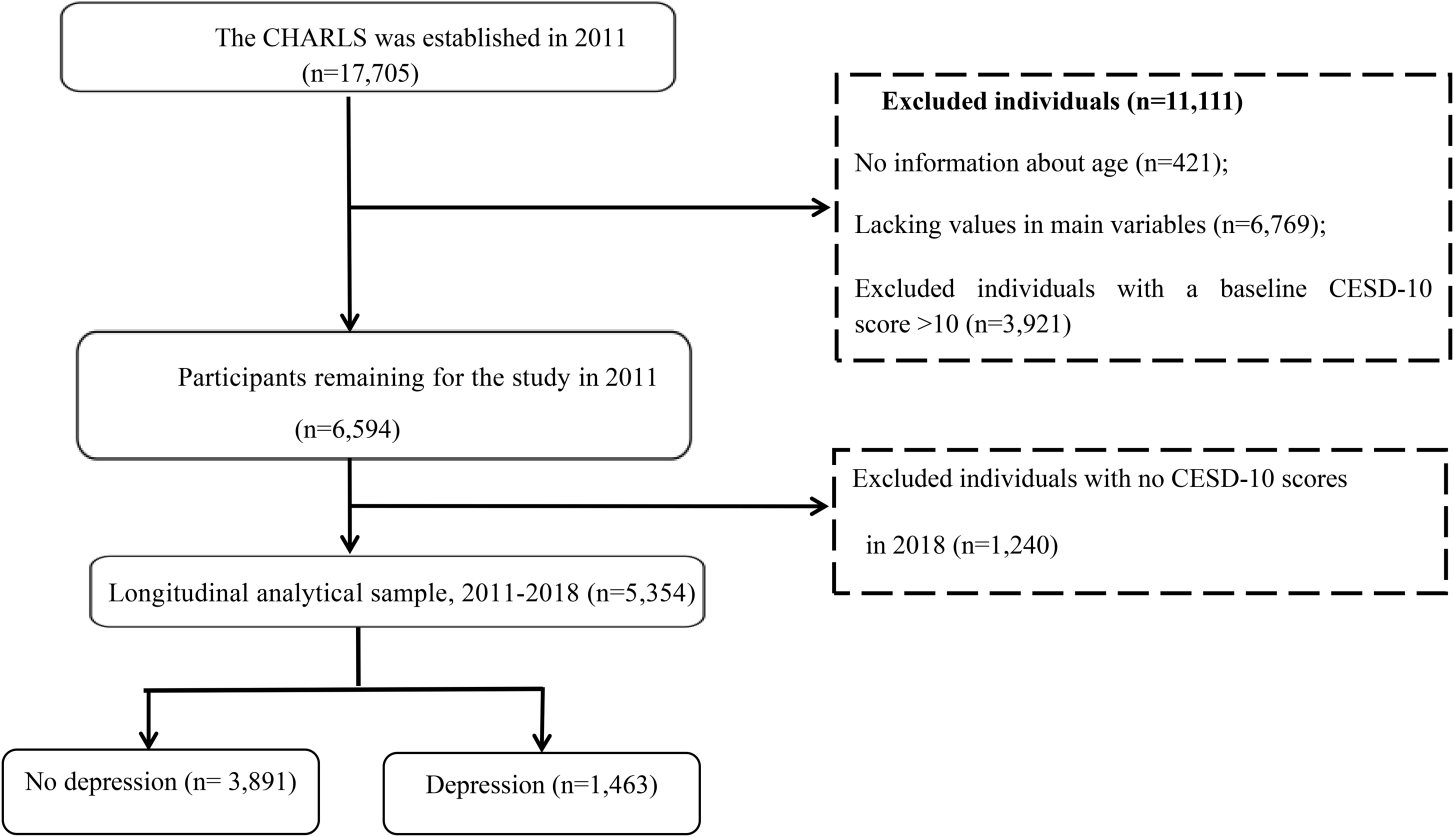
The study flowchart.

On the basis of these inclusion criteria, 6,594 participants were selected from among 17,705 participants aged 45 years or older; after excluding 421 participants with no information about age, 6,769 participants lacking values for the main variables and 3,921 participants with depressive symptoms at baseline were excluded. In the longitudinal analysis, we further excluded 1,240 participants without information on the CESD-10 scores in the CHARLS 2018. Our final analytic sample included 5,354 participants who had no depressive symptoms in the CHARLS 2011 and were followed up in 2018. The detailed selection process is shown in **Figure 1**.

The data were verified and entered by two trained researchers, and a database was built via EpiData 3.1 software (EpiData - Comprehensive Data Management and Basic Statistical Analysis System, EpiData Association, Odense, Denmark). The data were then double-checked.

### 2.2 Measures

#### 2.2.1 Exposure

The altitude is the altitude at which the participants lived in this study area. The altitudes were divided as follows: lower altitude: < 500 masl; middle altitude: 500-999 masl; high altitude: 1,000-1,499 masl; and higher altitude: ≥ 1,500 masl.

#### 2.2.2 Depressive symptoms

At baseline, cohort members were assessed with the CESD-10 to assess depressive symptoms in the CHARLS, which has been validated with older people in China (30). The participants were asked about the number of days they experienced every item during the previous week, such as feeling bothered, having trouble concentrating, feeling depressed, and feeling as though everything was effortful, feeling hopeful, feeling fearful, having restless sleep, feeling happy, feeling lonely, and having difficulty getting gone (31). Each item was rated on a 4-point Likert scale with answers varying from ‘rarely or none of the time (< 1 day)’ to ‘most or all of the time (5-7 days)’. The total score ranges from 0 to 30, with higher scores indicating more severe depressive symptoms (12). The CESD-10 used in this study exhibited good internal consistency (12). Previous studies have shown that a cutoff point of 10 has reasonable levels of sensitivity and specificity in older Chinese individuals (30). In our study, a subject who had a CESD-10 score greater than 10 was defined as having depressive symptoms.

### 2.3 Covariates

Our covariates included sex, age, marital status, education, professional status, residence, smoking status, alcohol consumption, height, and weight. Sex was divided into men and women. Age was divided into four groups: < 50 years, 50-59 years, 60-69 years, and ≥ 70 years. Marital status was divided into five groups: married and cohabiting, married but separated, separated or divorced, widowed, and never married. Education was divided into four groups: primary school or below, junior and senior high school, secondary and junior colleges, and bachelor’s degree or above. Residence was divided into rural areas and urban areas. The profession was divided into farmer, enterprise, self-employed, and others.

Health situation factors were measured as follows. BMI (body mass index) was obtained as the weight (kg) divided by the square of the height (m). We categorized BMI into four groups: underweight, BMI < 18.5 kg/m^2^; normal weight, BMI 18.5-24.99 kg/m^2^; overweight, BMI 25-29.99 kg/m^2^; and obese, BMI ≥ 30 kg/m^2^ (32). Other factors included smoking status (never, still smoking, quit smoking) and alcohol consumption (never, less than once a month, and more than once a month).

### 2.4 Statistical analyses

The R Project for Statistical Computing (R-4.2.1-win, University of Science and Technology of China; 2022-06-23) was used for the statistical analyses. Categorical data are presented as frequencies and percentages. Continuous data conforming to a normal distribution are presented as the means and standard deviations, and two independent samples t tests or chi-square tests were used for comparisons between groups. We used univariate analysis to determine the factors associated with depressive symptoms. Multivariate logistic regression was subsequently used to assess the associations between depressive symptoms and altitude among the participants. P values < 0.05 were considered to indicate statistical significance.

## 3 Results

### 3.1 Demographic characteristics of the participants and depressive symptoms

The baseline characteristics of the participants in the longitudinal study are shown in **Table 1**. The sample had the following characteristics: the participants ranged in age from 45 to 88 years; the average age of the participants was 57 (IQR: 50-63) years; 2,829 (52.8%) of the participants were men, and 2,525 (47.2%) were women; 3,274 (61.2%) of the participants had completed primary school or below; 4,731 (88.4%) individuals were married and cohabiting; 4,334 (80.9%) individuals lived in rural areas; the average BMI of the participants was 23.54 (IQR: 21.3-26.14) kg/m^2^; 3,253 (60.8%) individuals had a normal weight; the average altitude of the participants was 170 (IQR: 69-500) masl; and 3,992 (74.6%) individuals were at a lower altitude (**Table 1**).

**Table 1:** Demographic characteristics of the participants and their depressive symptoms.

Variables	Overall n (%)	Non depressive symptoms n (%)	depressive symptoms n (%)	P
Total number	5,354	3,891 (72.7)	1,463 (27.3)	
Sex				< 0.001
Men	2,829 (52.8)	2,192 (77.5)	637 (22.5)	
Women	2,525 (47.2)	1,699 (67.3)	826 (32.7)	
Age, y (median [IQR])	57 (50,63)	57 (50,63)	56 [50,62.5]	0.390
Age groups, y				0.447
< 50	,1242 (23.2)	890 (71.7)	352 (28.3)	
50-59	2,121 (39.6)	1,536 (72.4)	585 (27.6)	
60-69	1,514 (28.3)	1,123 (74.2)	391 (25.8)	
≥ 70	477 (8.9)	342 (71.7)	135 (28.3)	
Education				< 0.001
Primary school or below	3,274 (61.2)	2,275 (69.5)	999 (30.5)	
Junior and senior high school	1835 (34.3)	1408 (76.7)	427 (23.3)	
Secondary and junior colleges	223 (4.2)	189 (84.8)	34 (15.2)	
Bachelor’s degree or above	22 (0.4)	19 (86.4)	3 (13.6)	
Marital status				0.115
Married and cohabiting	4,731 (88.4)	3,459 (73.1)	1,272 (26.9)	
Married but separated	208 (3.9)	146 (70.2)	62 (29.8)	
Separated or Divorced	45 (0.8)	33 (73.3)	12 (26.7)	
Widowed	349 (6.5)	242 (69.3)	107 (30.7)	
Never married	21 (0.4)	11 (52.4)	10 (47.6)	
Profession status				< 0.001
Farmer	4,101 (76.6)	2,904 (70.8)	1,197 (29.2)	
Enterprise	868 (16.2)	711 (81.9)	157 (18.1)	
Self-employed	142 (2.7)	100 (70.4)	42 (29.6)	
Others	243 (4.5)	176 (72.4)	67 (27.6)	
Residence				< 0.001
Rural areas	4,334 (80.9)	3,101 (71.6)	1,233 (28.4)	
Urban areas	1,020 (19.1)	790 (77.5)	230 (22.5)	
BMI, kg/m^2^ (median [IQR])	23.54 [21.3,26.14]	23.56 [21.32,26.14]	23.47 [21.22,26.13]	0.309
BMI groups				0.013
Underweight	227 (4.2)	148 (65.2)	79 (34.8)	
Normal weight	3,253 (60.8)	2,372 (72.9)	881 (27.1)	
Overweight	1,549 (28.9)	1,148 (74.1)	401 (25.9)	
Obesity	325 (6.1)	223 (68.6)	102 (31.4)	
Smoking				< 0.001
No	3,089 (57.7)	2,168 (70.2)	921 (29.8)	
Still smoking	1,788 (33.4)	1,362 (76.2)	426 (23.8)	
Quit smoking	477 (8.9)	361 (75.7)	116 (24.3)	
Alcohol consumption				< 0.001
Never	3,374 (63)	2,368 (70.2)	1,006 (29.8)	
Less than once a month	452 (8.4)	338 (74.8)	114 (25.2)	
More than once a month	1,528 (28.5)	1,185 (77.6)	343 (22.4)	
Altitude, masl (median [IQR])	170 (69,500)	162 [58,481]	203 [78.5,668]	< 0.001
Elevation groups				< 0.001
Lower altitude	3,992 (74.6)	2,950 (73.9)	1,042 (26.1)	
Middle altitude	563 (10.5)	405 (71.9)	158 (28.1)	
High altitude	250 (4.7)	170 (68)	80 (32)	
Higher altitude	549 (10.3)	366 (66.7)	183 (33.3)	

Lower altitude, < 500 masl; middle altitude, 500-999 masl; high altitude, 1,000-1,499 masl; higher altitude, ≥ 1,500 masl.

Underweight, BMI < 18.5 kg/m^2^; normal weight, BMI 18.5-24.99 kg/m^2^;

overweight, BMI 25-29.99 kg/m^2^; and obesity, BMI ≥ 30 kg/m^2^.

The characteristics of the participants with different degrees of depressive symptoms are shown in **Table 1**. A total of 1,463 (27.3%) participants had depressive symptoms after follow-up. There were statistically significant differences between participants with different degrees of depressive symptoms across the following variables: sex, education level, professional status, residence, BMI, smoking status, alcohol consumption, and altitude (P < 0.05). The following factors were associated with a greater incidence of depressive symptoms among participants: women, primary school education or below, self-employed, rural area, underweight, no smoking, never alcohol consumption, and higher altitude.

### 3.2 Logistic regression analysis of the influencing factors of altitude and depressive symptoms in the participants

**Figure 2:**
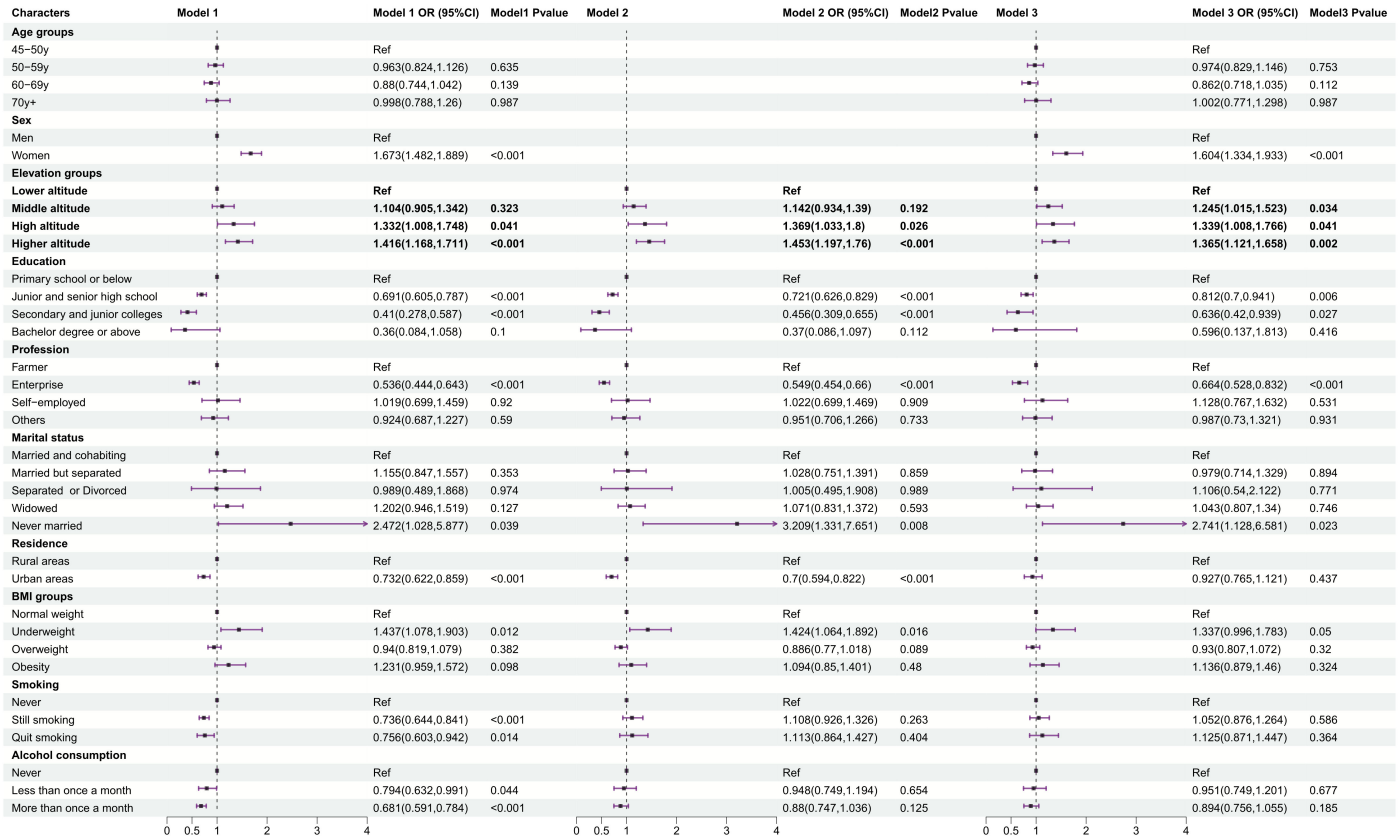
Logistic regression analysis of the influencing factors of altitude and depression in the participants. Lower altitude, < 500 masl; middle altitude, 500-999 masl; high altitude, 1,000-1,499 masl; higher altitude, ≥ 1,500 masl. Model 1: univariate logistic regression; Model 2: multivariate logistic regression adjusted for age and sex;Model 3: multivariate logistic regression adjusted for all other relevant variables (P<0.05) (Model 3, **Figure 2**).

Univariate logistic regression analysis was used to analyze the influencing factors of depressive symptoms and demonstrated that sex, altitude, education level, professional status, marital status, residence, BMI, smoking status, and alcohol consumption were the main risk factors for depressive symptoms among participants (P < 0.05) (Model 1, **Figure 2**). Multivariate logistic regression adjusted for sex and age revealed that altitude, education level, professional status, marital status, residence, and BMI remained statistically significant (P < 0.05) (Model 2, **Figure 2**). Multivariate logistic regression adjusted for all other relevant variables showed that sex, altitude, education level, professional status, and marital status remained statistically significant (P < 0.05) (Model 3, **Figure 2**). The following factors were associated with a greater incidence of depressive symptoms: female, middle altitude, high altitude, higher altitude, junior and senior high school, secondary and junior college primary school or below, enterprise and never married.

### 3.3 Marginal effects of altitude conditioned by related exposure characteristics and sex on depressive symptom scores

**Figure 3:**
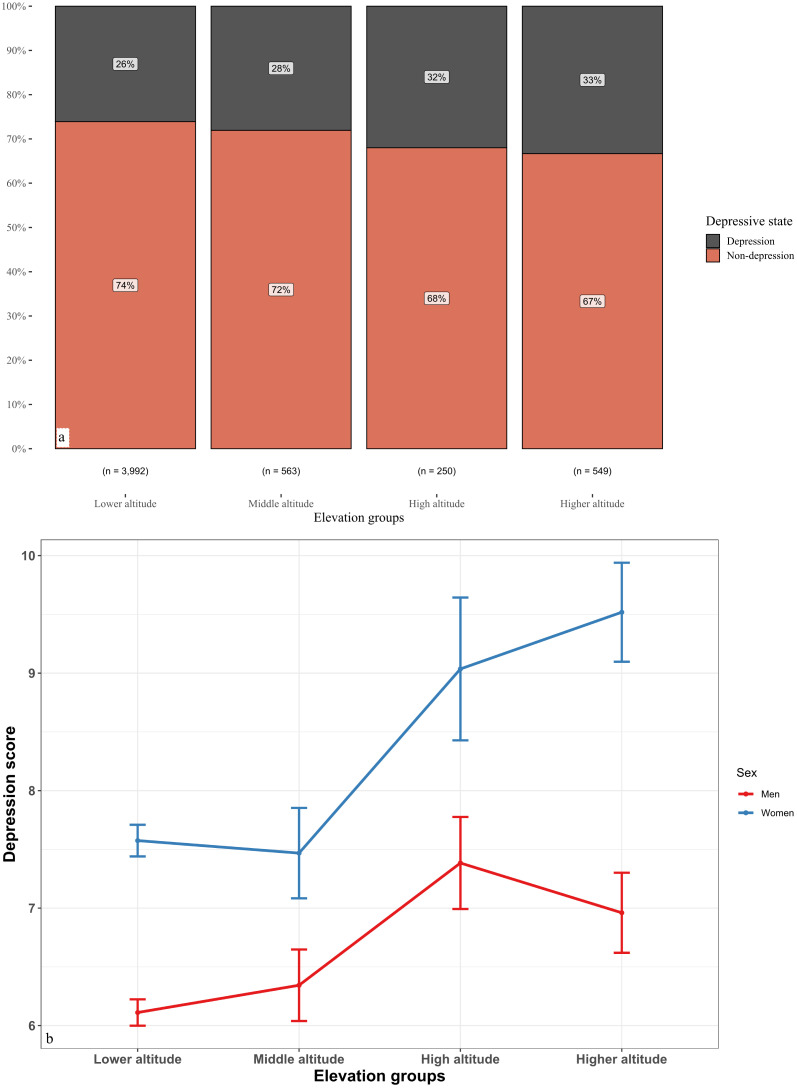
Marginal effects of altitude conditioned by related exposure characteristics and sex on depressive symptom scores.

The results revealed that at altitudes < 500 masl, 500–999 masl, 1,000–1,499 masl, and > 1,500 masl, the prevalence of depressive symptoms was 20%, 28%, 32% and 33%, respectively (**Figure 3A**). The results revealed that women’s depressive symptom scores were greater than men’s depressive symptom scores and that a greater altitude corresponded to greater women’s depressive symptom scores (**Figure 3B**). Men’s depressive symptom scores increased from low altitude to high altitude but decreased from high altitude to higher altitude (**Figure 3B**).

## 4 Discussion

This study adopted the CESD-10 to conduct the first longitudinal cohort survey of depressive symptom levels and altitude among middle-aged and elderly community-dwelling adults in China. The high prevalence of depressive symptoms among participants living at high altitudes may be due to differences in demographic characteristics and chronic hypoxia due to the high altitude during follow-up. After adjusting for possible confounders, we found that sex, altitude, education level, professional status and marital status were likely risk factors for depressive symptoms in participants.

This study suggested that the prevalence of depressive symptoms in individuals was 27.3%, which is lower than that reported in previous Chinese studies (28.6%) (16) and higher than that reported by other Chinese scholars (4.1% to 16.5%) (31, 32), a global prevalence of 17.9% (33) and a Spanish study (7.12% to 7.23%) (34). This is due to environmental conditions specific to the region that limit access to mental health services for individuals living in these areas and physiological mechanisms that exacerbate depressive symptoms (16, 35). However, our data revealed a linear relationship between altitude and depressive symptom risk; that is, the greater the altitude was, the greater the prevalence of depressive symptoms. This result is consistent with that of another study in Peru (35) but inconsistent with the findings of Japanese scholars (36). We found that when the altitude is more than 1,500 masl, the prevalence of depressive symptoms may increase by 1.365 times. The differences in the results may be explained by basic demographics, interpersonal relationships, lifestyles, education levels, and religious beliefs (36).

China has two different geographical regions (plains and mountains) and 56 ethnic groups. The fact that most people live in plain areas less than 500 masl, with high levels of economic development and social support, may play an important protective role against depressive symptoms. However, the poor geographical environment, lack of basic resources and facilities, low population density, and lack of social support at high altitudes make it difficult to obtain information on people’s health status (16, 37), and a variety of factors affect the development of depressive symptoms. Therefore, people living at high altitudes with depressive symptoms should be treated in a comprehensive manner that takes into account the geographical conditions and biological changes associated with living in these areas that may aggravate depressive symptoms (38).

Our findings are consistent with those of Moreno-Agostino et al. That is, there was a significant increase in the likelihood of depressive symptoms over time within populations, which did not seem to be explained by study design differences or publication bias alone (39).

The altitude of residence is a risk factor that has been identified in previous studies as being associated with the development of depressive symptoms (23, 35, 38). There are several possible reasons for this finding. First, low oxygen concentrations induce changes in monoaminergic neurotransmitters (mainly serotonin synthesis and signaling pathways) and brain structures involved in changes in cognitive function and inflammatory changes that lead to an increase in the number of cells that make up the immune system, which are associated with the pathophysiology of depressive symptoms and their severity (19, 40, 41). Second, it is related to the climate and geographical characteristics of the high-elevation area. These regions are characterized by cold climates, low humidity, strong winds, and altitude (which affects daylight hours), which alters hippocampal neurogenesis, reduces serotonin bioavailability, alters circadian cycles, and negatively affects melatonin secretion, which has been linked to depressive symptoms (42, 43). Third, people who live at high altitudes have low concentrations of folate and other micronutrients in their food and are prone to depressive symptoms (44). Therefore, medical personnel should consider biological and inflammatory characteristics when performing psychological and pharmacological interventions in populations living at high altitudes to reduce fatal consequences such as suicide (38).

The associations of other variables with the incidence of depressive symptoms are worth highlighting in our study. For example, being a woman (OR: 1.604, 95% CI: 1.334-1.933), being never married (OR: 2.741, 95% CI: 1.128-6.581) and being an enterprise (OR: 0.664, 95% CI: 0.528, 0.832) are directly proportional to the presence of depressive symptoms, whereas the degree of education is inversely related. High levels of education are associated with a lower likelihood of developing depressive symptoms, and our findings are consistent with those of previous studies (45, 46). This may be because people with higher education levels have greater knowledge in various fields and are able to actively cope with stress and negative emotions in life and work, which leads to a lower prevalence of depressive symptoms, which is inconsistent with previous research results (16). In contrast, participants living at high altitudes and with low education levels were unable to win the stress and negative emotions of work and life due to the influence of useless thoughts in reading, coupled with medical and economic conditions, which may increase depressive symptoms.

This study revealed that women had a greater prevalence of depressive symptoms than men did, which is consistent with the results of many previous studies (16, 35, 47). A study by Suradom et al. reported a common genetic predisposition to anxiety and depressive symptoms, a theory that has long been investigated with the belief that genetic variation in personality traits could play a role in the development of depressive symptoms (48). Women are generally more emotional than men are, are more likely to become excited or impulsive, have large mood swings, and are less adaptable, resulting in more negative emotions and high levels of depressive symptoms (16). Therefore, attention should be given to individual gender differences in depressive symptoms to develop targeted interventions for adults at high altitudes.

This study revealed that participants who lived with their original partner had a lower prevalence of depressive symptoms than did those who were married but were separated, separated or divorced, widowed, or never married. We found that the prevalence of depressive symptoms was 2.741 times greater among never-married participants than among those with other marital statuses. This difference may be related to the fact that they had a stable and relatively stable family harmonious home environment. A good family environment may play an important role in inhibiting the development of depressive symptoms (16).

Our study also revealed that the prevalence of depressive symptoms in enterprises was significantly greater than that in self-employed individuals and others. This finding is inconsistent with other reports (16). This may be because, with China’s economic transformation and rapid development, compared with self-employed individuals and others, enterprise employees may face more stressful events, which can contribute to depressive symptoms.

Moreover, in this study, underweight participants appeared to be more likely to experience depressive symptoms than were normal-weight, overweight or obese participants (P = 0.05). In contrast to Western perceptions, being thin can reflect poor nutrition, poor health or even poverty in China (31). Being underweight may affect a person’s body satisfaction and self-esteem and increase risk factors for cardiovascular disease (49). Chronic diseases and the cost of care, as well as inadequate care and poor doctor-patient relationships, increase the likelihood of depressive symptoms; this finding is consistent with those of previous studies (12, 31, 50). That is, people with chronic diseases are at greater risk of being depressed or suffering from depressive symptoms.

No supporting evidence was found for alcohol consumption and smoking in middle-aged and older adults being associated with depressive symptoms in China. These results are in sharp contrast to those of previous studies (21, 50, 51). In China, the primary motivation for drinking is to ward off the cold in high-altitude areas. Thus, alcohol consumption and smoking were not associated with depressive symptoms in our study. Depressive symptom levels were not significantly different across residences in our study. This result is consistent with previous research (16). This may be related to the high prevalence and poor recognition of depressive symptom in middle-aged and older adults in China (8).

Our cohort findings confirmed an increase in the prevalence of depressive symptoms over time, and these findings should be used as a basis for identifying at-risk populations. In this way, the coverage of prevention and treatment measures can be extended to high-altitude areas of China, where access to health services is scarce. In addition, oxygen-related treatments, such as hyperbaric oxygen therapy, 5-HTP therapy and ventilation techniques, can prevent the development of depressive symptoms and other mental disorders (51, 52). Therefore, the information provided in this study supports the need for clinical research on the prevention and treatment of depressive symptoms at high altitudes.

## 5 Limitations

There are several limitations to this study. This cohort study was conducted from the CHARLS 2011 to 2018 among middle-aged and older adults in China. The causal relationship between altitude and depressive symptoms could be clarified; however, there are still limitations. First, given the nature of the study, possible confounding factors that may influence the presence of depressive symptoms (such as religious affiliation, ethnicity, violence and migration) were not assessed. However, a study by Ishikawa et al. indicated that cultural factors such as religious outlook and social/family relationships inhibit the development of depressive symptoms in the Himalayas (25). Thus, cross-cultural studies could provide additional context and help determine whether the observed relationships hold true across different cultural settings and altitudes. Second, owing to self-reported data, the reliance on self-reported data, particularly for depressive symptoms assessed via the CESD-10 scale, may lead to underreporting or inaccuracies due to stigma or recall bias. This limitation is common in similar studies but can be mitigated by incorporating objective measures or corroborating reports from family members or healthcare providers in the future (53). However, surveyors have been trained, and the CESD-10 has been used in China. Therefore, we believe that these factors should not affect our results. Third, another study by O’Hare et al. (42) revealed that environmental conditions such as seasonal changes and pollution levels can significantly impact depressive symptom rates; thus, these factors should be considered in future research. Fourth, the inclusion of broader indicators of mental health, such as anxiety or cognitive decline, would provide a more complete picture of the psychological effects of living at high altitude (19).

## 6 Conclusions

In summary, this cohort study focused on the relationship between altitude and depressive symptoms among middle-aged and older adults in the Chinese community, preliminary to the discovery that middle-aged and older adults in the Chinese community had more depressive symptoms and a greater relationship with altitude. The factors affecting depressive symptoms in middle-aged and older adults are complex, and the prevalence of depressive symptoms increases with increasing altitude. This result should be tested in future follow-up studies with larger populations and over longer periods of time, including studies on unmeasured physiological, lifestyle and environmental variables. In this way, the role of hypoxia at low pressure at high altitudes on brain function could be supported by stronger evidence and could ultimately prevent depressive symptoms in at-risk people.

## Data Availability

The original contributions presented in the study are included in the article/supplementary material. Further inquiries can be directed to the corresponding author.
